# Lifetime co-occurrence of violence victimisation and symptoms of psychological ill health: a cross-sectional study of Swedish male and female clinical and population samples

**DOI:** 10.1186/s12889-015-2311-3

**Published:** 2015-09-28

**Authors:** Johanna Simmons, Barbro Wijma, Katarina Swahnberg

**Affiliations:** Department of Acute Internal Medicine and Department of Clinical and Experimental Medicine, Linköping University, Linköping, Sweden; Department of Clinical and Experimental Medicine, Linköping University, Linköping, Sweden; Department of Health and Caring Sciences, Faculty of Health and Life Sciences, Linnaeus University, Kalmar, Sweden

**Keywords:** Abuse, Mental health, Stress, Intimate partner violence, Re-victimisation, Poly-victimisation, Cumulative violence

## Abstract

**Background:**

Lifetime co-occurrence of violence victimisation is common. A large proportion of victims report being exposed to multiple forms of violence (physical, sexual, emotional violence) and/or violence by multiple kinds of perpetrators (family members, intimate partners, acquaintances/strangers). Yet much research focuses on only one kind of victimisation. The aim of this study was to investigate the association between symptoms of psychological ill health, and A) exposure to multiple forms of violence, and B) violence by multiple perpetrators.

**Method:**

Secondary analysis of cross-sectional data previously collected for prevalence studies on interpersonal violence in Sweden was used. Respondents were recruited at hospital clinics (women *n* = 2439, men *n* = 1767) and at random from the general population (women *n* = 1168, men *n* = 2924). Multinomial regression analysis was used to estimate associations between exposure to violence and symptoms of psychological ill health.

**Results:**

Among both men and women and in both clinical and population samples, exposure to multiple forms of violence as well as violence by multiple perpetrators were more strongly associated with symptoms of psychological ill health than reporting one form of violence or violence by one perpetrator. For example, in the female population sample, victims reporting all three forms of violence were four times more likely to report many symptoms of psychological ill health compared to those reporting only one form of violence (adj OR: 3.8, 95 % CI 1.6–8.8). In the male clinical sample, victims reporting two or three kind of perpetrators were three times more likely to report many symptoms of psychological ill health than those reporting violence by one perpetrator (adj OR 3.3 95 % CI 1.9–5.9).

**Discussion:**

The strong association found between lifetime co-occurrence of violence victimisation and symptoms of psychological ill-health is important to consider in both research and clinic work. If only the effect of one form of violence or violence by one kind of perpetrator is considered this may lead to a misinterpretation of the association between violence and psychological ill health. When the effect of unmeasured traumata is ignored, the full burden of violence experienced by victims may be underestimated.

**Conclusion:**

Different kinds of victimisation can work interactively, making exposure to multiple forms of violence as well as violence by multiple perpetrators more strongly associated with symptoms of psychological ill health than any one kind of victimisation alone.

## Background

Recently, there has been a growing understanding that different kinds of violence co-occur [1–3]. Many victims of interpersonal violence report being exposed to more than one form of violence (e.g., physical, sexual or emotional violence) and/or violence from more than one kind of perpetrator (e.g., family members, intimate partners, or peers) [1, 2, 4, 5]. In this study we investigate how this co-occurrence of violence for male and female victims is associated with symptoms of psychological ill health.

Different, but related concepts for describing the co-occurrence of violence have evolved. *Re-victimisation* focuses on victimisation across developmental periods, i.e., in both childhood and adulthood [4, 6]. In research on childhood abuse, *poly-victimisation* is used and relates to the number of incidents of victimisation [7]. Other terms, e.g., *multiple traumatic experiences, lifetime trauma,* and *cumulative abuse* also exist, but no consensus has evolved as to what concepts should be used in what contexts [1]. In this study, we use “*multiple forms of violence*” when referring to lifetime experiences of being subjected to more than one form of violence and “*violence by multiple perpetrators”* when referring to lifetime experiences of being subjected to violence by more than one kind of perpetrator. *"Co-occurrence of violence"* is used as an overriding term, including experiences of multiple forms of violence and/or violence by multiple perpetrators.

Associations between exposure to violence and psychological ill health have been found repeatedly. Both re-victimisation and poly-victimisation are more strongly associated with depression and anxiety than any kind of victimisation alone [6–9]. Poly-victimisation has been described as living in a violent condition where stressful experiences of victimisation pile up, leaving the victim at greater risk of developing symptoms of depression and anxiety [7, 9]. In this study it is suggested that exposure to multiple forms of violence and violence by multiple perpetrators can have a similar impact on victims.

The underlying mechanisms leading from violence to psychological ill health are multifactorial and not fully understood. An altered biological stress response has been postulated to be one of the mediating links [1, 10, 11]. Chronic stress can lead to alterations in the hypothalamic-pituitary-adrenal axis (HPA-axis) affecting secretion of the stress hormone cortisol. Changes have been found among female victims of physical and sexual violence as well as among patients with depression and Post Traumatic Stress Disorder [10]. It is likely that victims of multiple forms of violence and/or violence by multiple perpetrators suffer from a chronic alteration of the stress response to a greater extent than do victims of a single violent event, leading to a higher risk of psychological ill health.

**Fig. 1:**
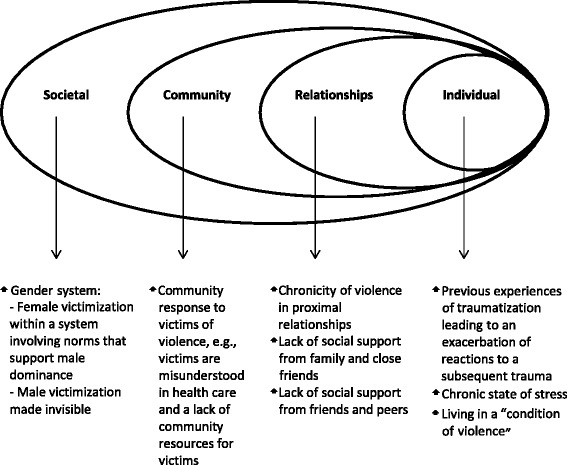
The ecological framework in which violence is conceptualized and understood in the present study. Examples given on each level are used to understand why victims of multiple forms of violence and/or violence by multiple perpetrators could be especially at risk of developing psychological ill-health

In this study, violence is conceptualized within an ecological framework in which factors on individual, relational, community and societal levels are considered [12]. In research on intimate partner violence, the ecological model has been used to reveal explanatory factors concerning why violence occurs [13, 14]. We use the model to understand why victims of multiple forms of violence and/or violence by multiple perpetrators could be especially at risk of developing psychological ill health (Fig. 1). At the individual level, experiences of fear, betrayal and helplessness can lead to an exacerbation of the previously victimised individual’s reaction to subsequent trauma [15]. At the relational level, violence in proximal relationships, between partners, or within a family, has the worst effect on victims’ psychological health [16–18]. One explanation for this could be the chronic nature of such violence, with victims and perpetrators being bound together in a continuous relationship [13]. Violence within families may even be intergenerational: children witnessing interparental violence are at greater risk of being exposed to intimate partner violence in adulthood [19]. Also, violence within a family may lead to family breakdown, which can have negative health effects on children, whether or not they are subjected to violence themselves. At the relational and community levels, positive family resources and support from peers as well as from the school system are known to be important factors for developing resilience among children [20]. Such support is likely to be less for victims of multiple forms of violence and/or violence by multiple perpetrators, e.g., re-victimised women are at higher risk of living with low social support than are women victimised as children or as adults [21]. Also community resources and the health care response to victims of violence have been found to be inadequate [22–24].

At the societal level, the gender system affects the conceptualization of violence: men are generally considered to be perpetrators and women victims. Consequently, male victimisation is not as well understood as female victimisation. Ehrensaft and colleagues report an association between being subjected to violence by a partner, and depression, anxiety or PTSD for female but not male victims [25]. Likewise, Sundaram and colleagues found an association between physical violence from any perpetrator, and self-reported symptoms of depression among women, but not among men [26]. However, others report an association between intimate partner violence and symptoms of both mood and anxiety disorders for female as well as male victims [27], but in some cases found the association to be stronger for women [28, 29]. Though rarely investigated in adult samples including both men and women, co-occurrence of violence has, in a few studies, been associated with depression and anxiety in both sexes [30, 31]. However, one difficulty when comparing psychological health effects between the sexes is that men and women are often reported to be exposed to forms of violence that are conceptually different. For example, intimate partner violence against men and against women is markedly different regarding context, chronicity, and severity [29, 32–34], and in some, studies co-occurrence of violence among adults has been reported to be more common among female victims than among male victims [28, 31, 35].

The co-occurrence of violence has previously been examined in the same samples as is used in this study. It was found that 47–48 % of female and 29–31 % of male victims reported experiences of multiple forms of violence, and 33–37 % of female and 22–23 % of male victims reported violence by multiple perpetrators [5]. This is in line with a growing body of research suggesting that co-occurrence of violence is common. Some have even suggested that exposure to violence by multiple perpetrators is the norm rather than the exception [2]. However, important research fields such as intimate partner violence, childhood abuse, community violence and workplace violence are still, to a large extent, separate. The resulting fragmented focus on one kind of violent behaviour, or violence from one kind of perpetrator may lead to misinterpretation of the relationship between violence and psychological ill health. Adverse health outcomes that should have been attributed to the cumulative effect of several forms of violence and/or violence by more than one kind of perpetrator may be incorrectly attributed to a single kind of victimisation. It has been suggested that disregarding the co-occurrence of violence introduces a significant bias in studies of interpersonal violence [2, 36].

The aim of this study was to investigate how lifetime experiences of multiple forms of violence (model 1) and violence by multiple perpetrators (model 2) were associated with self-reported symptoms of psychological ill health among men and women in clinical and population samples in Sweden. We also put forward the following hypothesis:

Being subjected to both A) multiple forms of violence (model 3) and B) violence from multiple perpetrators (model 4), would be more strongly associated with symptoms of psychological ill health than reporting exposure to one form of violence/violence from one kind of perpetrator.

## Method

### Procedure

This study is based on secondary analyses of data collected in four previous prevalence studies of exposure to interpersonal violence among men and women in Sweden. Detailed descriptions of the data collection have been published previously [37–40]. The population samples consist of women (*n* = 1166 response rate = 61%) and men (*n* = 2924, response rate = 50 %) recruited at random from a county population in the years 1999 and 2001 (female sample) and 2007 (male sample). The clinical samples consist of 2439 women (response rate = 81 %) recruited from gynaecology clinics at three hospitals in the years 1999 to 2000, and 1766 men (response rate = 74 %) recruited from six somatic clinics at one hospital in 2005. In the population samples, being between the age of 18 and 64 was an inclusion criterion. In the clinical sample no upper age limit was used, resulting in an age range of 18 to 88 (female clinical sample) and 18 to 91 (male clinical sample).

### Measures

The NorVold Abuse Questionnaire (NorAQ) was used to operationalize violence and to estimate self-reports of symptoms indicating psychological ill health [41, 42]. Questions about emotional, physical and sexual violence, as well as symptoms of psychological ill health and background characteristics are addressed.

The test-retest reliability for the questions concerning symptoms of psychological ill health ranged between 70–89 % for women and 66–80 % for men. Corresponding figures for the questions concerning violence were 84–95 % for women and 77–100 % for men. The questions concerning violence in NorAQ have been validated in male and female samples, using a face-to face interview as the gold standard. The results of the validation have been published previously [41, 42]. For women the positive likelihood ratio was 38 for emotional, 6 for physical and 42 for sexual violence, while the corresponding figures for men were 3 for emotional, 9 for physical and 46 for sexual violence. The questions about violence in NorAQ can be found in Table 1. One question about mild physical violence was also included but it had low concurrent validity and was therefore excluded from the present study. Respondents who answered ‘yes’ to that question but ‘no’ to the subsequent questions about moderate and severe violence were considered non-victims of physical violence.

**Table 1:** Questions about exposure to interpersonal violence in NorAQ

Emotional violence	
Mild	Have you experienced anybody systematically and for a long period trying to repress, degrade, or humiliate you?
Moderate	Have you experienced anybody systematically and by threat or force trying to limit your contact with others or totally control what you may and may not do?
Severe	Have you experienced living in fear because somebody systematically and for a long period threatened you or somebody close to you?
Physical violence	
Moderate	Have you experienced anybody hitting you with his/her fist(s) or with a hard object, kicking you, pushing you violently, giving you a beating, thrashing you, or doing anything similar to you?
Severe	Have you experienced anybody threatening your life by, for instance, trying to strangle you, showing a weapon or knife, or by any other similar act?
Sexual violence	
Mild	Has anybody against your will touched parts of your body other than the genitals in a "sexual way" or forced you to touch other parts of his or her body in a "sexual way"?
Mild/sexual humiliation	Have you in any other way been sexually humiliated; for example, by being forced to watch a pornographic movie or similar against your will, forced to participate in a pornographic movie or similar, forced to show your body naked, or forced to watch when somebody else showed his/her body naked?
Moderate	Has anybody against your will touched your genitals, used your body to satisfy him/herself sexually, or forced you to touch anybody else’s genitals?
Severe	Has anybody against your will put his penis into your vagina, mouth or rectum or tried any of this, or put in or tried to put an object or other part of the body into your vagina, mouth or rectum?

Note: The word “vagina” was omitted from the male version of the questionnaire

Because the aim of the original data collections was not to investigate respondents’ health, no validated measurement of any diagnosis such as PTSD or depression was included in the data collection. For the analysis here, six items included in NorAQ were summarized and the resulting measurements should not be understood as diagnostic of any disease but rather as self-reports of symptoms indicating psychological ill health among victims. Three questions had a general character and asked if respondents, during the previous 12 months, for a long time and to such an extent that they found it difficult to cope with their daily life, had suffered from 1) anxiety, 2) depression, 3) insomnia. Three questions concerned symptoms of PTSD and asked if respondents during the previous 12 months had: 4) experienced unpleasant recollections that disturbed them and that they could do nothing about; 5) avoided situations in order not to have unpleasant recollections or feelings, and whether this had interfered with what they wanted to do; 6) felt as if their feelings were numb for a long period. Possible answers to each question were: a) No; b) Yes, but rarely; c) Yes sometimes; and d) Yes, often. To test if the questions pertained to one underlying construct, an explanatory factor analysis was conducted. One factor was found, explaining 59.5 % of the variance in the questions about symptoms of psychological ill health. The answer to each question was therefore given a score ranging from zero for “No” to three for “Yes, often”, resulting in a sum score with the range 0–18 (Cronbach’s alpha = 0.86). The resulting score was highly positively skewed and residuals were not normally distributed; hence, linear regression analysis could not be used. Instead respondents were categorised depending on their score in the following categories: 1) No symptoms of psychological ill health (0 points): 55 % of respondents; 2) Few symptoms (1–6 points): 36 %; and 3) Many symptoms (7–18 points): 9 % of respondents. The cut-off between “few symptoms” and “many symptoms” was chosen because seven points necessitated that the respondents were given a score of two on at least one of the questions.

In NorAQ, two to four different questions are used for each form of violence to discriminate between mild, moderate and severe acts of violence (Table 1). However, this fragmentation is arbitrary and does not consider the context of victimisation or the victim-perpetrator relationship, e.g., within a specific context a “mild” act of violence might have a severe impact on victims. In this study the different degrees of severity of violence were considered rather as different manifestations of each form of violence, and therefore the different degrees of severity were merged. By doing so, it was also possible to investigate exposure to multiple forms of violence by creating one variable, where respondents were grouped according to their lifetime experiences of exposure to different forms of violence: 1. no violence; 2. physical; 3. emotional; 4. sexual; 5. emotional and physical; 6. emotional and sexual; 7. physical and sexual; 8. emotional, physical and sexual.

Respondents were asked who had subjected them to each form of violence, and were given a list of nine different alternatives. Three groups were then created depending on the victim-perpetrator relationship: 1. family (parents, step parents and/or sibling); 2. partner (former and/or present partner); 3. acquaintance/stranger (same-age playmate, schoolmate or other person under 18, a known person who did not belong to your family, a person totally unknown to you and/or other). To investigate the effect of being exposed to violence by multiple perpetrators, one variable was created where the respondents were grouped as follows; 1. no violence, hence no perpetrator 2. family 3. partner 4. acquaintance/stranger 5. family and partner 6.family and acquaintance/stranger 7. partner and acquaintance/stranger 8. family, partner and acquaintance/stranger.

#### Statistical analyses

The statistical software SPSS, version 20, was used to compute all analyses. Pearson’s chi square test was used to test for differences in background characteristics between the sexes in the population and clinical samples respectively (Table 2).

**Table 2:** Background characteristics of respondents

	Population samples				Clinical samples			
	Women *n* = 1166		Men *n* = 2924		Women *n* = 2439		Men *n* = 1766	
	*n*	%	*n*	%	*n*	%	*N*	%
Age group	*P* = 0.03				*p* < 0.01			
≤29	251	21.8	594	20.5	390	16.1	114	6.5
30-39	241	20.9	562	19.4	523	21.5	143	8.1
40-49	284	24.6	648	22.4	489	20.1	174	9.9
≥50	377	32.7	1090	37.7	1027	42.3	1329	75.5
Civil status	*p* < 0.01				*p* < 0.01			
Single	224	19.4	839	29.2	374	15.5	356	20.3
Partner	931	80.6	2035	70.8	2045	84.5	1401	79.7
Education	*p* = 0.3				*p* < 0.001			
≤12 years	644	55.5	1668	57.4	1526	62.9	1198	68.2
≥13 years	517	44.5	1237	42.6	901	37.1	558	31.8
Occupation	*P* < 0.01				*p* < 0.001			
Employed	807	70.8	2308	80.0	1599	67.7	825	46.9
Retired, sick- leave, social welfare	106	9.3	196	6.8	427	18.1	840	47.8
Other (Student, Unemployed, pregnant or parental leave)	227	19.9	383	13.3	337	14.3	93	5.3

Note: Item non-response n = 16-84 (0.4-2 %). P values represent differences in the distribution of background characteristics between the sexes in the clinical and population samples respectively

Sex-segregated analyses were performed for both clinical and population samples separately. First, multinomial regression analysis was used to investigate the association between symptoms of psychological ill health (response variable) and exposure to multiple forms of violence (model 1) as well as violence by multiple perpetrators (model 2). “No symptoms of psychological ill health” was used as the reference category for the response variable. In the male clinical sample, experiences of multiple forms of violence including sexual violence as well as violence by family and partner perpetrators were only reported by a few respondents. For this reason these categories had to be collapsed and the explanatory variables were restricted to include information about whether respondents reported one or more forms of violence (model 1) and one or more kinds of perpetrators (model 2). All models were adjusted for age-group, educational level, civil state and current occupation.

To test the hypothesis that exposure to A) multiple forms of violence (model 3) and B) violence by multiple perpetrators (model 4) were more strongly associated with symptoms of psychological ill health than one form of violence/violence by one kind of perpetrators, multinomial regression analyses were again used. Analyses were performed among the sub-set of respondents who reported some kind of violence. “Symptoms of psychological ill health” was used as the response variable, and as explanatory variables, information about the number of different forms of violence (model 3) and number of different kinds of perpetrators (model 4) was used.

### Ethics

Answering questions about experiences of violence and victimisation might elicit feelings of distress and anxiety among respondents. For some men and women, simply receiving the questionnaire might have triggered negative reactions and flashbacks. Therefore, everyone receiving NorAQ was invited to contact either an independent therapist or the research team if they wanted. However, this was an option used by very few. The study was approved by the regional ethical review board in Linköping, Sweden (registration no 37–07).

## Results

Background characteristics of the samples are presented in Table 2. Men were more often single than women and more likely to be over 60 years old. Especially in the clinical samples, the men were considerably older and also more often retired or on sick leave than were the women (Table 2).

Models 1 and 2 showed that for both men and women and in both clinical and population samples, reporting multiple forms of violence (Tables 3 and 4) as well as violence by multiple kinds of perpetrators (Table 5 and 6) were more strongly associated with reporting psychological ill health than reporting one form of violence or violence by one kind of perpetrator. Tables 3–6 also indicate that the specific kind of violence and the victim-perpetrator relationship is important. In both sexes, emotional violence and violence by an intimate partner, or any of the combinations including these kinds of violence, had higher OR for reporting symptoms of psychological ill health than other forms of violence/violence by other perpetrators.

**Table 3:** The association between exposure to multiple forms of violence and symptoms of psychological ill-health in the population samples (Model 1)

		Women					Men				
			Symptoms of psychological ill-health					Symptoms of psychological ill-health			
			Few (score =1-6)		Many (score 7–18)			Few (score =1-6)		Many (score 7–18)	
			*N* = 454 (40.7 %)		*N* =112 (10.0 %)			*N* = 883 (31.5 %)		*N* = 217 (7.7 %)	
		*N*	OR	95 % CI	OR	95 % CI	*N*	OR	95 % CI	OR	95 % CI
Civil state	Partner	901	1		1		1982	1		1	
	Single	215	1.2	0.8–1.6	1.5	0.9–2.6	819	1.4	1.2–1.8	2.3	1.6–3.2
Education	≥13	501	1		1		1194	1		1	
	<12	615	0.8	0.6–1.1	0.9	0.6–1.5	1607	0.8	0.7–0.99	0.8	0.6–1.1
Occupation	Employed	787	1		1		2251	1	-	1	
	Retired, sick-leave social welfare	104	2.8	1.7–4.7	7.7	3.8–15.6	187	2.0	1.3–2.9	14.9	9.2–24.2
	Other	225	1.4	0.98–2.0	1.8	0.99–3.3	363	1.4	1.03–1.8	3.0	1.9–4.9
Agegroup	<29	248	1		1		567	1		1	
	30–39	234	0.8	0.5–1.3	1.4	0.7–2.7	550	1.1	0.8–1.4	1.0	0.6–1.7
	40–49	279	0.8	0.5–1.1	0.7	0.3–1.3	625	0.9	0.7–1.2	1.5	0.9–2.5
	>50	355	0.8	0.6–1.2	0.7	0.3–1.4	1059	0.7	0.5–0.9	0.8	0.5–1.3
Form of violence	No violence	711	1		1		1619	1		1	
	Emotional	77	3.0	1.7–5.3	12.9	5.9–28.3	153	3.2	2.2–4.6	8.2	4.5–14.9
	Physical	77	2.9	1.7–4.8	3.8	1.4–10.2	624	1.5	1.2–1.8	2.5	1.6–3.8
	Sexual	63	2.0	1.1–3.5	5.8	2.4–14.0	40	1.6	0.8–3.1	1.9	0.4–8.9
	Emo + Phys	60	5.1	2.5–10.4	27.8	11.6–66.8	266	4.8	3.5–6.6	21.2	13.3–33.8
	Emo + Sex	33	4.0	1.6–9.9	18.0	5.9–55.1	14	1.4	0.4–5.0	4.9	0.98–24.5
	Phys + Sex	19	2.2	0.9–5.8	2.3	0.3–19.1	24	2.4	1.003–5.5	4.1	0.8–20.5
	Emo + Phys + Sex	76	5.7	2.8–11.4	28.6	12.3–66.1	61	12.1	5.0–29.4	72.6	27.2–193.8
Model fit:		R^2^ = 0.20 (Cox & Snell), 0.24 (Nagelkerke).					R^2^ = 0.19 (Cox & Snell), 0.24 (Nagelkerke).				
		Model *χ* ^2^(28) = 252.87					Model *χ* ^2^(28) = 600.76				

Note: Reference category is “no symptoms of psychological ill-health” (score 0).Emo= Emotional violence, Phys = Physical violence, Sex= Sexual violence. Occupation “other”=Unemployed, student, pregnant or parental leave

**Table 4:** The association between exposure to multiple forms of violence and symptoms of psychological ill-health in the clinical samples (Model 1)

		Women						Men				
			Symptoms of psychological ill-health						Symptoms of psychological ill-health			
			Few (score =1–6)		Many (score 7–18)		No of forms of violence*		Few (score =1–6)		Many (score 7–18)	
			N = 922 (40.3 %)		N =222 (9.7 %)				N = 560 (33.1 %)		N = 125 (7.4 %)	
		*N*	OR	95 % CI	OR	95 % CI		*N*	OR	95 % CI	OR	95 % CI
Civil state	Partner	1932	1		1			1349	1			
	Single	356	1.2	0.9–1.5	1.6	1.1–2.4		341	1.4	1.03–1.8	2.0	1.2–3.1
Education	≥13	873	1		1			539	1			
	<12	1415	1.0	0.8– 1.2	1.2	0.8–1.7		1151	1.2	0.97–1.6	1.4	0.9–2.3
Occupation	Employed	1562	1		1			793	1			
	Retired, sick-leave social welfare	404	1.4	1.03–1.8	4.3	2.8–6.6		806	1.1	0.8–1.4	2.3	1.4–3.8
	Other	322	1.1	0.8–1.5	1.8	1.1–2.8		91	1.4	0.8–2.4	4.7	2.0–10.9
Agegroup	<29	373	1		1			107	1			
	30–39	512	0.8	0.6–1.1	0.9	0.5–1.5		138	0.7	0.4–1.3	2.1	0.7–6.3
	40–49	469	0.8	0.6–1.1	0.9	0.5–1.5		170	1.0	0.5–1.8	4.7	1.6–13.5
	>50	934	0.6	0.5–0.8	0.5	0.3–0.8		1275	0.6	0.4–1.02	1.4	0.5–3.9
Form of violence	No violence	1527	1		1		No violence	1103	1			
	Emotional	106	2.4	1.5–3.8	10.0	5.4–18.6	One form	418	1.9	1.5–2.4	3.1	1.9–5.0
	Physical	166	1.8	1.3–2.6	4.1	2.3–7.3	≥Two forms	169	5.1	3.4–7.6	19.6	11.3–34.2
	Sexual	129	1.5	1.1–2.3	4.2	2.2–8.0						
	Emo + Phys	107	3.2	2.0–5.2	12.8	6.9–24.0						
	Emo + Sex	60	7.1	3.3–15.3	26.7	10.8–66.1						
	Phys + Sex	54	1.9	1.02–3.5	7.6	3.4–17.1						
	Emo + Phys + Sex	139	8.8	4.8–16.1	53.9	27.4–106.0						
Model fit:		R^2^ = 0.17 (Cox & Snell), 0.20 (Nagelkerke).						R^2^ = 0.13 (Cox & Snell), 0.15 (Nagelkerke).				
		Model *χ* ^2^(28) = 423.69						Model *χ* ^2^(18) = 228.17				

Note: Reference category is “no symptoms of psychological ill-health” (score 0). Emo = Emotional violence, Phys = Physical violence, Sex = Sexual violence. Occupation “other” = Unemployed, student, pregnant or parental leave

**Table 5:** The association between violence by multiple perpetrators and symptoms of psychological ill-health in the population sample (Model 2)

		Women					Men				
			Symptoms of psychological ill-health					Symptoms of psychological ill-health			
			Few (score =1–6)		Many (score 7–18)			Few (score =1–6)		Many (score 7–18)	
			*N* = 454 (40.6 %)		*N* =112 (10.0 %)			*N* = 883 (31.5 %)		*N* = 217 (7.7 %)	
		*N*	OR	95 % CI	OR	95 % CI	*N*	OR	95 % CI	OR	95 % CI
Civil state	Partner	902	1		1		1982	1		1	
	Single	215	1.1	0.8–1.6	1.5	0.9–2.6	819	1.4	1.2–1.7	2.3	1.6–3.2
Education	≥13	501	1		1		1194	1		1	
	<12	616	0.8	0.6–1.05	0.9	0.6–1.5	1607	0.8	0.7–0.99	0.8	0.6–1.1
Occupation	Employed	788	1		1		2251	1		1	
	Retired, sick-leave social welfare	104	2.8	1.6–4.7	8.2	4.0–16.6	187	1.9	1.3–2.9	15.3	9.5–24.6
	Other	225	1.5	1.04–2.1	1.9	1.1–3.4	363	1.4	1.04–1.8	3.1	1.9–4.9
Agegroup	<29	248	1		1		567	1		1	
	30–39	234	0.8	0.5–1.3	1.2	0.6–2.5	550	1.0	0.8–1.4	1.0	0.6–1.7
	40–49	279	0.8	0.5–1.2	0.6	0.3–1.3	625	0.8	0.6–1.1	1.4	0.8–2.4
	>50	356	0.8	0.6–1.3	0.6	0.3–1.2	1059	0.7	0.5–0.9	0.7	0.4–1.1
Kind of perpetrator	No violence	712	1		1		1619	1		1	
	Family	58	3.3	1.8–6.2	7.0	2.7–18.4	102	2.1	1.4–3.4	7.0	3.6–13.9
	Partner	72	4.8	2.7–8.6	7.7	3.0–20.0	35	6.2	2.5–15.2	34.5	12.2–97.6
	Community	125	1.8	1.2–2.7	7.9	4.1–15.3	779	2.0	1.7–2.4	3.9	2.7–5.8
	Fam + Part	28	5.4	1.7–17.1	46.6	13.3–163.0	11	1.7	0.4–7.3	9.2	1.8–47.0
	Fam + Com	46	3.0	1.5–6.3	14.2	5.6–36.0	171	2.4	1.7–3.5	7.9	4.5–13.9
	Part + Com	44	5.7	2.5–12.9	16.5	5.7–48.2	50	4.9	2.4–10.1	26.8	11.2–64.4
	Fam + Part + Com	32	9.1	2.6–31.9	53.2	13.7–206.2	34	4.5	1.7–12.4	54.4	19.3–152.8
Model fit:		R^2^ = 0.21 (Cox & Snell), 0.24 (Nagelkerke).					R^2^ = 0.17 (Cox & Snell), 0.21 (Nagelkerke).				
		Model *χ* ^2^(28) = 258.36					Model *χ* ^2^(28) = 521.31				

Note: Reference category is “no symptoms of psychological ill-health” (score 0). Fam = Family, Part = partner, Acq/Str = Aquaintance/Stranger. Occupation “other” = Unemployed, student, pregnant or parental leave

**Table 6:** The association between violence by multiple perpetrators and symptoms of psychological ill-health in the clinical sample (Model 2)

		Women						Men				
			Symptoms of psychological ill-health						Symptoms of psychological ill-health			
		*N*	Few (score =1-6)		Many (score 7–18)		No of kinds of perpetrators*	*N*	Few (score =1-6)		Many (score 7–18)	
			*N* = 924 (40.3 %)		*N* =222 (9.7 %)				*N* = 561 (33.5 %)		*N* = 125 (7.4 %)	
Civil state	Partner	1935	1		1			1351	1		1	
	Single	357	1.2	0.9–1.5	1.7	1.1–2.5		342	1.4	1.03–1.8	2.0	1.3–3.1
Education	≥13	873	1		1			539	1		1	
	<12	1419	1.0	0.9–1.3	1.2	0.9–1.7		1154	1.2	0.98–1.6	1.5	0.9–2.3
Occupation	Employed	1563	1		1			795	1		1	
	Retired, sick-leave social welfare	407	1.4	1.05–1.8	4.5	3.0–6.9		807	1.0	0.8–1.3	4.7	2.1–10.5
	Other	322	1.1	0.9–1.5	1.8	1.1–2.8		91	1.4	0.8–2.3	2.2	1.4–3.7
Agegroup	<29	373	1		1			107	1		1	
	30–39	512	0.8	0.6–1.1	0.8	0.5–1.4		138	0.7	0.4–1.3	2.1	0.7–6.4
	40–49	470	0.8	0.6–1.1	0.8	0.5–1.4		170	1.0	0.5–1.7	4.8	1.7–13.6
	>50	937	0.6	0.5–0.8	0.4	0.3–0.7		1278	0.6	0.3–0.96	1.3	0.5–3.5
Kind of perpetrator	No violence	1531	1		1		No violence	1106	1		1	
	Family	123	2.1	1.4–3.1	3.3	1.6–6.6	One kind	449	2.1	1.7–2.7	4.0	2.6–6.3
	Partner	180	2.9	2.0–4.2	10.9	6.5–18.4	≥2 kinds	138	3.5	2.3–5.3	13.6	7.7–24.3
	Community	212	2.0	1.5–2.8	6.6	4.0–10.9						
	Fam + Part	44	3.6	1.6–8.0	17.7	7.1–44.0						
	Fam + Com	67	2.9	1.6–5.2	14.9	7.1–31.3						
	Part + Com	79	2.8	1.5–5.0	19.2	9.8–37.8						
	Fam + Part + Com	56	8.7	3.3–22.6	57.4	20.6–159.9						
Model fit:		R^2^ = 0.16 (Cox & Snell), 0.19 (Nagelkerke).						R^2^ = 0.11 (Cox & Snell), 0.14 (Nagelkerke).				
		Model *χ* ^2^(28) = 393.96						Model *χ* ^2^(18) = 199.49				

Note: Reference category is “no symptoms of psychological ill-health” (score 0)

Fam = Family, Part = Partner, Acq/Str = Aquaintance/Stranger. Occupation “other” = Unemployed, student, pregnant or parental leave

*Because the number of men subjected to some of the combinations of perpetrators was low in the male clinical sample, analyses could not be made for all possible combinations of perpetrators. Rather respondents were categorized according to the number of kinds of perpetrators they reported

When testing hypotheses A (model 3, Table 7) and B (model 4, Table 8) the importance of the quantitative aspects of victimisation was confirmed. For both sexes and in both samples, reporting multiple forms of violence was more strongly associated with reporting many symptoms of psychological ill health than reporting one form of violence. For example: in the female population sample, victims reporting experiences of all three forms of violence were almost four times as likely to also report”many symptoms” of psychological ill health (score 7–18) (adj OR 3.8, 95 % CI 1.6–8.8) (Table 7).

**Table 7:** Hypotheses A, testing if exposure to multiple forms of violence is more strongly associated to symptoms of psychological ill-health, than reporting one form of violence (model 3)

Number of forms of violence	Women							Men						
	Symptoms of psychological ill-health							Symptoms of psychological ill-health						
	*N*	Few (score 1–6)			Many (score 7–18)			*N*	Few (score 1–6)			Many (score 7–18)		
		OR	95 % CI		OR	95 % CI			OR	95 % CI		OR	95 % CI	
Population samples														
One form	217	1			1			817	1			1		
Two forms	112	1.5	0.8	2.6	2.4	1.2	4.9	304	2.5	1.8	3.4	5.5	3.6	8.6
Three forms	76	2.1	1.0	4.4	3.8	1.6	8.8	61	7.3	3.0	18.0	23.5	8.9	62.5
*Model fit*	*R* ^*2*^ *= 0.13 (Cox & Snell), 0.15 (Nagelkerke). Model χ* ^*2*^ *(18) = 54.73*							*R* ^*2*^ *= 0.21 (Cox & Snell), 0.24 (Nagelkerke). Model χ* ^*2*^ *(18) = 272.67*						
Clinical samples														
One form	401	1			1			418	1			1		
Two forms	221	1.8	1.2	2.7	2.4	1.5	4.0	169	2.7	1.8	4.2	6.3	3.5	11.1
Three forms	139	4.9	2.6	9.2	10.1	5.1	20.3							
*Model fit*	*R* ^*2*^ *= 0.14 (Cox & Snell), 0.16 (Nagelkerke). Model χ* ^*2*^ *(18) = 116.66*							*R* ^*2*^ *= 0.14 (Cox & Snell), 0.16 (Nagelkerke). Model χ* ^*2*^ *(18) = 89.00*						

Note: Reference category is “no symptoms of psychological ill-health” (score 0). All models are adjusted for age, educational leveL, civil state and occupation. Reference category is “no symptoms of psychological ill-health” = 0 points, “Low level “=1-6 points and High level is ≥7 points

**Table 8:** Hypotheses B, testing if exposure to violence by multiple perpetrators is more strongly associated to symptoms of psychological ill-health, than reporting violence from one kind of perpetrator (model 4)

	Women							Men						
	Symptoms of psychological ill-health							Symptoms of psychological ill-health						
Number of kinds of		Few (score 1–6)			Many (score 7–18)				Few (score 1–6)			Many (score 7–18)		
perpetrators	*N*	OR	95 % CI		OR	95 % CI		*N*	OR	95 % CI		OR	95 % CI	
Population samples														
One kind	255							916						
Two kinds	118	1.6	0.9	2.8	2.6	1.3	5.1	232	1.3	0.9	1.8	2.3	1.4	3.6
Three kinds	32	3.2	0.9	11.6	6.1	1.6	23.9	34	2.3	0.8	6.2	11.5	4.1	32.0
	*R* ^*2*^ *= 0.13 (Cox & Snell), 0.15 (Nagelkerke). Model χ* ^*2*^ *(18) = 56.00*							*R* ^*2*^ *= 0.16 (Cox & Snell), 0.18 (Nagelkerke). Model χ* ^*2*^ *(18) = 197.16*						
Clinical samples														
One kind	515							449						
Two kinds	190	1.3	0.9	2.0	2.5	1.6	4.1	138	1.7	1.1	2.6	3.3	1.9	5.9
Three kinds	56	3.8	1.4	10.2	8.5	3.1	23.8							
	*R* ^*2*^ *= 0.11 (Cox & Snell), 0.13 (Nagelkerke). Model χ* ^*2*^ *(18) = 89.95*							*R* ^*2*^ *= 0.10 (Cox & Snell), 0.11 (Nagelkerke). Model χ* ^*2*^ *(16) = 60.66*						

Note: Reference category is “no symptoms of psychological ill-health” (score 0). All models are adjusted for age, educational leveL, civil state and occupation

The same general pattern was found for reporting multiple perpetrators (Table 8). In both the male and female population and clinical samples, reporting exposure to violence by multiple perpetrators was more strongly associated with reporting many symptoms of psychological ill health compared to reporting one kind of perpetrator. For example: in the male population sample, men reporting two kinds of perpetrators were twice as likely to also report “many symptoms” of psychological ill health (score 7–18) (adj OR 2.3 95 % CI 1.4-3.6) than were men reporting one kind of perpetrator. However, there was generally no significant difference in the OR for reporting “few symptoms” of psychological ill-health (score 1–6) between victims reporting one kind of perpetrator and those reporting multiple kinds of perpetrators (Table 8).

## Discussion

### Co-occurrence of violence and symptoms of psychological ill health

Victims reporting all three forms of violence and/or violence by all three kinds of perpetrators had the highest likelihood of reporting symptoms of psychological ill health (model 1–2, Tables 3–6). This was in line with previous research and underlines the importance of the co-occurrence of violence [6, 21, 30, 31, 43]. It seems that different violent events work interactively, possibly creating a chronic state of stress. In accordance with this, prior studies have found that individuals who developed PTSD in response to a trauma are at increased risk of also suffering from PTSD after a subsequent trauma [15, 44]. Even merely stressful life events can have a sensitizing effect on victims, leading to PTSD after a lesser trauma that would not generally generate symptoms [45, 46]. This underlines the need to see different kinds of victimisation as integrated processes affecting victims over their entire lifetime. Among children and youth, poly-victimisation is described as living in a ‘violent condition’ rather than experiencing separate traumatic “events”. It is considered a condition where stressful experiences accumulate, increasing the risk of mental illness [7, 9]. It might be useful to adapt the same viewpoint for adult victims of multiple forms of violence, and violence by multiple perpetrators, and consider them also as living in a violent condition.

Though quantitative aspects of victimisation are important, so are qualitative aspects. The form of violence matters, as does the victim-perpetrator relationship. In this study, emotional violence was generally more strongly associated with symptoms of psychological ill health than other forms of violence (Tables 3 and 4). Also, violence from an intimate partner generally had the strongest association with self-reported symptoms of psychological ill health (Tables 5 and 6), a finding supported by some previous research [17]. However, though violence from an acquaintance/stranger showed the weakest association with symptoms of psychological ill health, it consistently contributed to higher OR when reported *in addition* to violence from other kinds of perpetrators. This is supported by previous findings that female victims of both community and partner violence reported more trauma symptoms than those reporting either community or partner violence alone [47]. When violence is part of both intimate relationships and more distant ones, victims might be deprived of all sanctuaries, which may impede the recovery process. For poly-victims compared to other victims, it has been hypothesized that when more environments as well as more people are associated with traumatic experiences, this may lead to more difficulties in resisting negative self-attribution [7].

### Gender

In this study, the same general pattern concerning the association between violence victimisation and symptoms of psychological ill health was found for men and women and in both clinical and population samples. It is possible that if we had been able to use another measurement of ill health, for example a diagnostic tool for PTSD or depression, or if we had included other kinds of victimisation in our study, gender differences concerning ill health would have emerged.

However, gender-based comparisons of the association between violence and psychological ill health should be made with caution. Men and women are exposed to different kinds of violence, and violence by different perpetrator*s.* As has also been reported by others, a previous descriptive study of the samples used in the current study found that men report a larger proportion of violence by acquaintances/strangers than women, and women report a larger proportion of intimate partner violence than men [5]. Because our measure of multiple forms of violence included violence by all kinds of perpetrators, the characteristics of violence reported by men and women are likely to be different, and gender-based comparisons of the risk of reporting symptoms of psychological ill health may be misleading. For this reason we have made a sex-segregated analysis.

One possible approach to this problem would be to conduct separate analyses of exposure to multiple forms of violence for victims of intimate partner violence, family violence, and violence by acquaintances/strangers. Indeed, this is the most common approach to investigating interpersonal violence. However, as has been described in detail for the current samples elsewhere, a substantial proportion of victims report more than one kind of perpetrator [5]. For example, as can be calculated from Table 5, in the population samples 176 women and 130 men reported some kind of intimate partner violence. Among those, 104 women (59 %) and 95 men (73 %) also reported violence perpetrated by a family member and/or an acquaintance/stranger. Similar findings of victimisation by multiple perpetrators have been reported by others [2]. Hence, if wanting to make an analysis for each sub-set of victims we would need to make analyses not only for intimate partner violence, family violence and violence by an acquaintance/stranger separately, but also for each possible combination of perpetrators. Perhaps the most interesting approach would be to include information both about exposure to multiple forms of violence and violence by multiple perpetrators in the same analysis, and investigate how those variables interact. However, such an analysis would require a larger data set than the present one.

The association between intimate partner violence and symptoms of psychological ill health was strong for both men and women. Previously, intimate partner violence against men has sometimes been reported to be associated with psychological ill health [27] and sometimes not [25]. Intimate partner violence against women occurs more frequently and is generally of a more severe nature than is intimate partner violence against men [32, 34]. However, some men are subjected to severe violence from their female intimate partners [48, 49]. Prevailing ideas of masculinity and a gendered cultural context portraying men as the perpetrators of violence and women as the victims shape the experiences and behaviours of victims, perpetrators and professionals [50]. However, the distinction between victim and perpetrator is not always clear. Many victims of intimate partner violence also report being the perpetrator of violence, and many violent incidents are bidirectional in nature [2, 3, 51, 52]. Also, violence occurs in both heterosexual and homosexual relationships. Hence, though violence should be conceptualized within a gender system involving norms that support male dominance over females, it is evident that both men and women can be both victims and perpetrators of violence. Our results, supported by some previous research, underline the fact that intimate partner violence against men exists, can be serious, and affects the psychological health of victims [27, 53]. In recent years the need for intervention programs for female victims of intimate partner violence has started to evolve [54]. This is important, but it is also critical that the health care system is individually tailored, both in respect to victims’ sex and to their lifetime history of violence from all kinds of perpetrators.

### Implications

We found that both exposure to multiple forms of violence and violence by multiple perpetrators were more strongly associated with symptoms of psychological ill health than reporting one form of violence/one kind of perpetrator (Tables 7 and 8). In previous studies, mainly concerning poly-victimisation, associations found between any single form of victimisation and psychological ill health have been found to be weakened or eliminated when poly-victimisation was included in the analysis [7, 9]. Only measuring the effect of one kind of violence or violence by one kind of perpetrator and ignoring the effect of unmeasured traumata as well as associations between different kinds of violence can introduce significant bias to a study. The relationship between violence and ill health can be misinterpreted, and the full burden of violence experienced by victims may thus be underestimated. To better understand the nature and consequences of exposure to interpersonal violence there is a need to find strategies and analytic methods to bridge the gaps between research focusing on specific kinds of violence, and to consider how different kinds of violence are intertwined and affect victims. Both reporting multiple forms of violence and violence by multiple perpetrators are common among victims and should therefore affect research methodology, treatment, and intervention programs [2, 8, 18, 35].

### Methodological considerations

This study was based on secondary analysis of previously collected data. The studies for which data was originally collected did not aim to investigate psychological ill health or the co-occurrence of violence specifically. As a consequence of this, some of the measurements used are problematic. Due to the construction of NorAQ, important types of victimisation such as witnessing violence or neglect of children or the elderly are not included. Also, we were not able to consider the frequency and duration of violence or the age at which victimisation occurred, nor could we separate reciprocal from non-reciprocal violence.

The results of this study underline the importance of incorporating different kinds of violence in the same study. However, there is no consensus on how to do this. In NorAQ, one question about the perpetrator follows after the questions covering each form of violence. Another possibility would have been to use perpetrator-specific questions. This difference may seem small, but has been found to significantly influence the results in research concerning intimate partner violence [55].

The proportion of victims suffering from violence by multiple perpetrators is underestimated in this study, as the number of perpetrators is not accounted for. Perpetrators were instead roughly classified into only three categories. This might also affect the association found between violence and symptoms of psychological ill health, since it is likely that being victimised by several perpetrators within one category has a stronger association with psychological ill health than being victimised by a single perpetrator. Also, NorAQ does not include any measurements of duration or frequency of violent acts, which might be important for the health outcome. However, the psychological health effects of co-occurring violence have been reported to be more dependent on reporting multiple forms of victimisation, which we do measure, rather than the duration or frequency of a specific trauma [9, 18]. Considering the strong association found between symptoms of psychological ill health and both exposure to multiple forms of violence and violence by multiple perpetrators, these deficits in our study further emphasize the need to use an integrated approach in study design and include as many kinds of victimisation as possible when researching violence.

Because the primary intention of the data collection was not to measure the respondents’ health, our measurement of symptoms indicating psychological ill health is questionable: the questions have not been validated by other means than test-retest. However, the results of that procedure were satisfactory. Also, the explanatory factor analysis implies one underlying construct and the results are in accordance with previous studies finding associations between being subjected to interpersonal violence and symptoms of depression, anxiety and PTSD [6, 28, 31]. For these reasons, we believe that our measurement of symptoms indicating psychological ill health is relevant, but scoring high on the measure should not be interpreted as diagnostic of any mental disorder.

The study design only allows for investigating associations; causality cannot be proven. As in other retrospective, cross-sectional studies the temporal sequence between exposure to violence and the onset of symptoms of psychological ill health is unknown and unmeasured confounders could possibly influence the associations found.

The data for this study was collected within a rather large time frame, but because our focus is on lifetime experiences we do not think it likely that this influenced the results in any major way. The response rate varied quite considerably between the samples (50–81 %) and was especially low in the male population sample. This is a threat to the generalizability of our results. However, our main finding, the association between symptoms of psychological ill health and exposure to multiple forms of violence violence/violence by multiple perpetrators, held true for both sexes and in both the clinical and the population samples. For this reason we believe that neither the timespan nor the response rate had a significant influence on the results.

## Conclusion

In this study we found that co-occurrence of violence victimisation is an important aspect of interpersonal violence. Both exposure to multiple forms of violence and violence by multiple perpetrators were more strongly associated with symptoms of psychological ill health than experiences of one kind of violence. This result was the same for both male and female victims in the clinical and population samples. Not considering this in research may lead to misinterpretation of the association between violence and symptoms of psychological ill health.
